# First International Precision Vaccines Conference: Multidisciplinary Approaches to Next-Generation Vaccines

**DOI:** 10.1128/mSphere.00214-18

**Published:** 2018-08-01

**Authors:** Francesco Borriello, Simon D. van Haren, Ofer Levy

**Affiliations:** a Precision Vaccines Program, Division of Infectious Diseases, Boston Children’s Hospital, Boston, Massachusetts, USA; b Harvard Medical School, Boston, Massachusetts, USA; c Department of Translational Medical Sciences, University of Naples Federico II, Naples, Italy; d Center for Basic and Clinical Immunology Research (CISI), University of Naples Federico II, Naples, Italy; e WAO Center of Excellence, Naples, Italy; f Broad Institute of MIT & Harvard, Cambridge, Massachusetts, USA; UMKC School of Medicine

**Keywords:** adjuvants, immunization, meeting report, precision vaccines, systems vaccinology

## Abstract

Vaccines represent a remarkable success in the history of medicine since they have prevented and, in some instances, eradicated a range of infectious diseases. However, for many existing vaccines, immunogenicity is limited, requiring multiple booster doses, and we are still unable to target many pathogens due to intrinsic features of the microorganism, such as genetic/antigenic variability between strains, and our limited understanding of the variables that regulate vaccine responsiveness, including age- and sex-specific differences. Moreover, the traditional approach to vaccine development is often empirical, relying on inactivation of microorganisms or purification of their components, which are usually less immunogenic than the whole microorganism from which they derive. This approach has yielded multiple important vaccines but has failed to consistently generate vaccines that are sufficiently immunogenic in populations with limited immune responsiveness such as newborns and elderly individuals. In an effort to trigger impactful collaborations, a community of scientists gathered in Boston in the United States for the first biennial International Precision Vaccines Conference, sponsored by the Boston Children’s Hospital Precision Vaccines Program, to discuss innovation in vaccinology. Recent advancements in the field of systems biology that can identify vaccine immunogenicity biomarkers for target populations, in human *in vitro* models, and in novel adjuvant and formulation strategies offer unprecedented opportunities to dissect the human immune response to vaccines and inform dramatic improvements in vaccine efficacy. These approaches are poised to have a major scientific and translational impact in vaccinology.

## Abstract (precis)

Vaccines represent a remarkable success in the history of medicine since they have prevented and, in some instances, eradicated a range of infectious diseases. However, for many existing vaccines, immunogenicity is limited, requiring multiple booster doses, and we are still unable to target many pathogens due to intrinsic features of the microorganism, such as genetic/antigenic variability between strains, and our limited understanding of the variables that regulate vaccine responsiveness, including age- and sex-specific differences.

## INTRODUCTION

Vaccines are among the most effective interventions available to prevent infectious diseases and represent a prominent success in the history of medicine (1). Nevertheless, several factors may impact and sometimes hamper responses to vaccine, including immune status (e.g., healthy versus immunocompromised subjects) (2). There are also sex-specific (i.e., male versus female) and age-specific (i.e., newborn/infant versus adult versus elderly) differences in immune responses (3–7) that impact vaccine efficacy. The importance of this concept is highlighted by the higher rate of death due to infections at the extremes of life than is seen with middle-aged adults (8). Of note, the distinct immune response of the newborn and the young infant over the first 6 months of life creates a “window of vulnerability” during which some vaccines may be less effective than later in life (9). Other factors that may influence immune responses and therefore vaccine efficacy are circadian and circannual rhythms as well as geographical location due to factors such as distinct genetic and epigenetic backgrounds (10, 11). These concepts should be taken into account in developing vaccines that target pathogens endemic to a specific geographical area. Finally, in considering vaccine (self-)adjuvantation, whole, live microorganisms activate distinct immune responses that are typically more robust than those induced by their components (12, 13). The use of optimized adjuvanted vaccine formulations targeted to a given population may overcome barriers in vaccine development and match or even exceed pathogens in eliciting effective immune responses (14).

**FIG 1:**
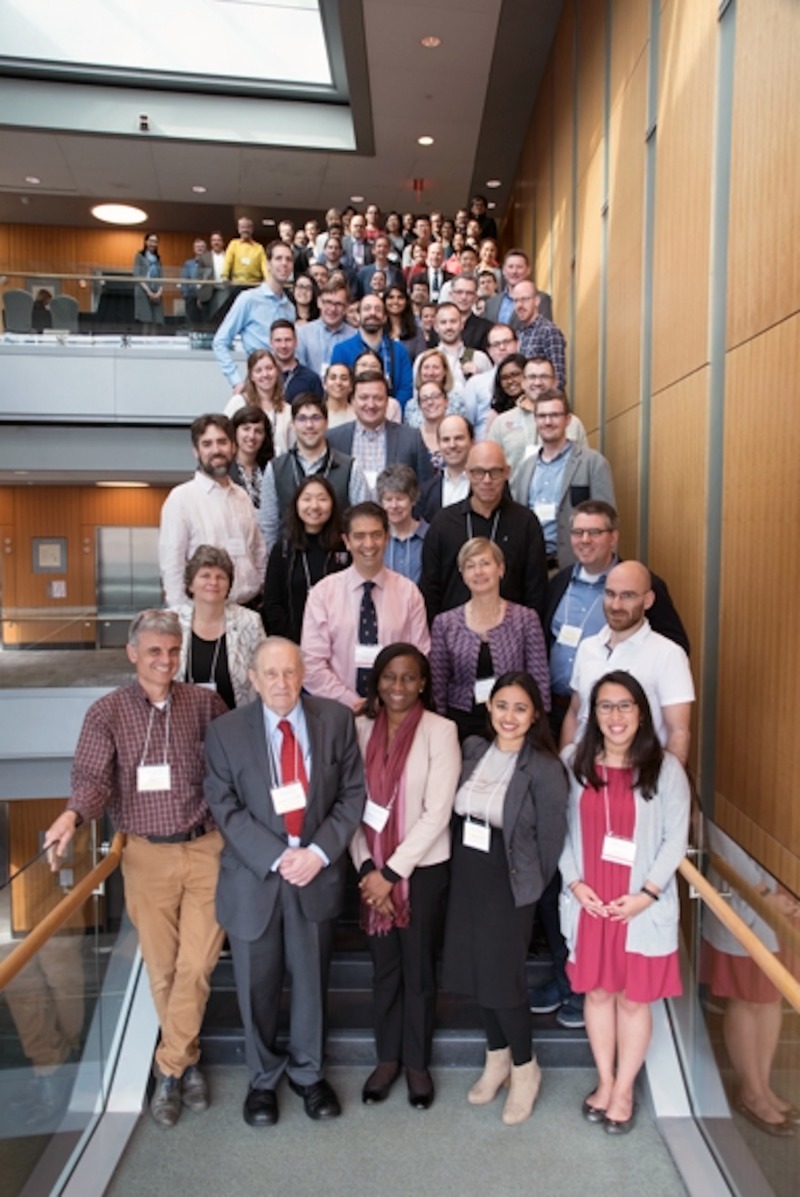
Attendees of the first biennial International Precision Vaccines Conference (23 and 24 October 2017), held at the Joseph B. Martin Conference Center at Harvard Medical School (Boston, MA, USA) and sponsored by the Boston Children’s Hospital Precision Vaccines Program (Boston, MA, USA).

To develop strategies and synergies in vaccine development, a community of experts in a range of fields, including immunology, pediatrics, vaccinology, systems biology, biostatistics, and bioinformatics as well as vaccine adjuvantation and formulation, gathered at the Joseph B. Martin Conference Center at Harvard Medical School (Boston, MA, USA) on 23 and 24 October 2017 for the first biennial International Precision Vaccines Conference (IPVC) (Fig. 1). The following sections report highlights of the conference oral presentations. The IPVC was hosted by Boston Children’s Hospital’s Precision Vaccines Program (http://www.childrenshospital.org/research-and-innovation/research/departments/medicine/precision-vaccines-program), whose aim is to promote collaboration in development of vaccines tailored to distinct vulnerable populations.

## PRECISION VACCINES PROGRAM

**FIG 2:**
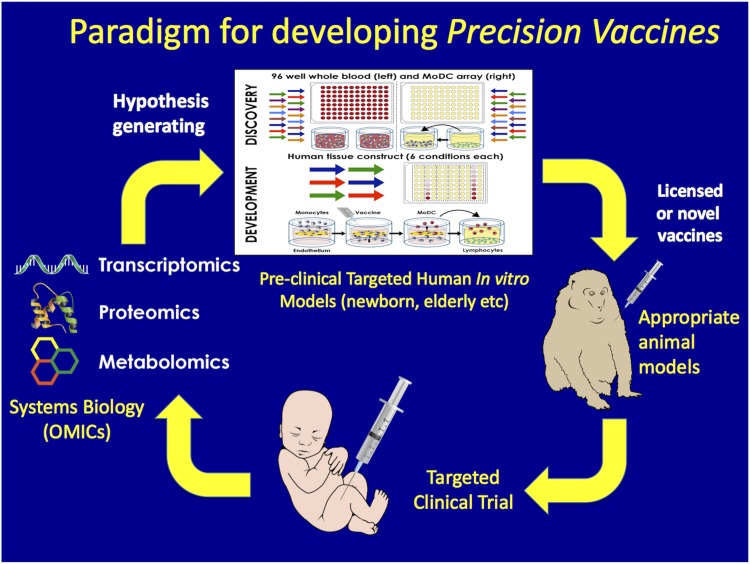
Paradigm for the cycle of precision vaccinology. Development of precision vaccines may include systems biology analysis of biosamples from clinical trials to define molecular pathways that correlate with immunogenicity, thereby generating new hypotheses; use of *in vitro* systems for hypothesis testing; and characterization of population-specific responses, thereby informing use of appropriate animal models and targeted clinical trials.

After brief welcoming remarks from Gary R. Fleisher (Department of Medicine, Boston Children’s Hospital and Harvard Medical School, Boston, MA, USA), Ofer Levy (Precision Vaccines Program, Boston Children’s Hospital and Harvard Medical School, Boston, MA, USA) introduced the Precision Vaccines Program that sponsored the conference. Based in the Division of Infectious Diseases at Boston Children’s Hospital, and comprised of >200 members from academia, government, private-public foundations, and industry, the Precision Vaccines Program is a platform for international collaboration to foster development of vaccines targeted at vulnerable populations such as the young and elderly. Recent advances in molecular and systems biology and genetics as well as in translational medicine have informed a precision medicine strategy of defining subpopulations of patients sharing similar characteristics and tailoring medical interventions according to a patient’s responsiveness (15). The use of this approach in vaccinology, further enhanced by advances in immune ontogeny and human *in vitro* culture systems as well as in adjuvantation and formulation science, is paving the way for the development of precision vaccines, defined as vaccines that (i) take into account the target population; (ii) are formulated to selectively activate the immune system by targeting anatomic sites, cells, and pathways that generate a protective response; and (iii) may, as needed, contain adjuvantation systems known to optimally enhance immunogenicity in a target population. In order to accomplish this complex and crucial task, systems biology methodologies such as transcriptomics, proteomics, and metabolomics as well as preclinical human *in vitro* models that take into account age- and sex-specific differences can be leveraged to generate hypotheses to be tested in appropriate animal models and eventually in targeted clinical trials (Fig. 2). Key to the success of this approach is interdisciplinary collaboration such as that catalyzed by the Precision Vaccines Program and Conference.

## HORIZON FOR NEW VACCINE DEVELOPMENT

Stanley A. Plotkin (University of Pennsylvania, Philadelphia, USA) outlined major obstacles in defining effective vaccine approaches for specific microorganisms and possible strategies to overcome them. Examples of these are influenza viruses, Bordetella pertussis, human immunodeficiency virus (HIV), rotavirus, and respiratory syncytial virus (RSV). The efficacy of influenza vaccines is hindered by influenza virus variability, and ways to improve it include the addition of adjuvants and/or of a conserved antigen such as stalk hemagglutinin (16, 17). A major limitation of the current acellular pertussis vaccine is its waning immunity (i.e., short effector memory immune response) compared to whole-cell pertussis vaccine. Therefore, the durability of immunity after pertussis vaccine can be limited, especially several years postimmunization. Approaches to improve the development of long-lasting Th1/Th17 and Tfh responses after pertussis immunization may include enhancing acellular pertussis vaccines by increasing the content of B. pertussis antigens and/or by addition of adjuvants. Alternatively, a genetically detoxified pertussis toxin or vaccines based on newer circulating strains could be employed (18–20). These approaches are not mutually exclusive, and all of them are likely to provide important information on the immune response to pertussis vaccines. Another major pathogen for which no approved vaccine exists is the human immunodeficiency virus (HIV). HIV rapidly disseminates from mucosal sites of infection and exhibits extreme strain variability, leading to the development of broadly neutralizing antibodies only late during the infection. A recent clinical trial highlighted the importance of antibody-dependent effector functions for protecting against HIV infection. Therefore, boosting these functions with appropriate vaccine vectors and/or adjuvants may represent a viable strategy for HIV vaccine development. Other promising approaches include the induction of broadly neutralizing antibodies through envelope trimer structures or effector CD8 T cells that kill infected cells using cytomegalovirus (CMV) vectors (21–26). Rotavirus represents an interesting example of population-specific challenges to vaccine development for which current vaccines show poor efficacy in children living in resource-poor countries in Asia and Africa. It is conceivable that the efficacy of rotavirus vaccines is affected by differences in gut microbiome composition, a parameter that needs to be considered in future clinical trials (27), as well as by breastmilk antibodies, concurrent oral polio vaccine, malnutrition, and myotonic dystrophy. Recent studies of RSV demonstrate how developments in structural biology can inform vaccine design. Modification of the RSV fusion (F) protein such that it retains its prefusion conformation is critical to the generation of protective neutralizing antibody responses (28, 29). More generally, molecular dissection of B cell and antibody repertoires in vaccinated or infected individuals coupled with structural biology has informed structure-based antigen design and is poised to define a new generation of vaccine candidates (30). Additional novel approaches that will likely impact vaccine development include nucleic acid-based vaccines, adjuvant molecules that target innate immune pathways in order to polarize the adaptive immune response and induce long-lasting memory, and innovative formulation strategies as well as exploitation of the beneficial role of heterologous (“nonspecific”) vaccine effects (31–34). In addition, systems biology tools will be key to define novel correlates of protection (35, 36).

## ONTOGENY

The session on ontogeny, the change in a given individual across time, considered how the developing early life immune system impacts infection risk and interventions aimed to enhance host defense as well as systems biology tools to study the immune system in early life. James Wynn (University of Florida, Gainesville, FL) highlighted the high incidence and mortality rates of sepsis early in life, especially in preterm newborns (37). In particular, the newborn demonstrates both quantitative and qualitative differences from the adult in nearly all aspects of immunity which at least partially explain increased susceptibility to infection. These differences, however, might reflect the need to curb the inflammatory response that may arise in the course of the transition of the newborn from the fetal environment to the external world. Nevertheless, the adaptability of the immune system early in life provides the potential for increasing host protection through a number of interventions, namely, maternal immunization, breastfeeding, newborn/infant immunization, and probiotics (4).

Tobias Kollmann (University of British Columbia, Vancouver, Canada) described how finding biomarkers for successful vaccination can provide useful information for the optimization of vaccines in which low or partial responsiveness is observed in early life. Challenges in biomarker identification include (i) uncertainty regarding which biomarkers to pursue (e.g., antibodies, innate or adaptive immune signatures) due to our limited knowledge of the underlying diseases and (ii) the fact that biomarkers that correlate with protection might not necessarily give insight into mechanisms of protection—i.e., they may not be mechanistic biomarkers. An unbiased approach using systems biology tools holds promise to define biomarkers of newborn vaccine immunogenicity (35, 36). However, most of the studies using these methodologies have been conducted in young adults due to the requirement in conventional protocols for relatively large volumes of biosamples (e.g., blood). To overcome this hurdle, the Expanded Program on Immunization Consortium (EPIC), an association of academic centers comprised of Boston Children’s Hospital (Boston, MA, USA), the University of British Columbia (Vancouver, Canada), Medical Research Council—The Gambia (Fajara, The Gambia), the Free University of Brussels (Brussels, Belgium), and the Telethon Kids Institute (Perth, Australia) is partnering to study pediatric systems vaccinology. Working with support from the National Institutes of Health (NIH) National Institute of Allergy & Infectious Diseases (NIAID) Human Immunology Project Consortium (HIPC), EPIC has established analytical and computational tools to conduct systems biology studies with less than 1 ml of blood, a volume easily obtained from newborns and infants. The EPIC-HIPC project is now taking advantage of this important “small sample-big data” technological advance to conduct systems biology analyses on the peripheral blood of human newborns immunized with hepatitis B vaccine (HBV) and Mycobacterium bovis bacillus Calmette-Guérin (BCG) vaccine to characterize the molecular ontogeny of the peripheral blood compartment in the first week of human life and then assess vaccine-induced perturbations of that ontogeny to define molecular signatures predicting immunogenicity.

## CLINICAL CONSIDERATIONS IN PRECISION VACCINOLOGY

Properly designed clinical trials represent the foundation of precision vaccinology efforts and are critical to advance the understanding of the early life immune system and unveil new therapeutic avenues for preventing early life infections. Beate Kampmann (Medical Research Council Unit, Fajara, The Gambia) described examples of how combining clinical trials and immunological studies can provide insights into the ontogeny of the immune response as well as into maternal factors shaping newborn risk of infection and response to vaccines. For example, in a cross-sectional study of *in vitro* Toll-like receptor (TLR) responses in Gambian infants across the first year of life, significant differences were noted between different TLR agonists, with TLR8 agonists demonstrating robust activation of Th1-polarizing cytokine induction in early life and between birth and 1 month of age (38). Analysis of levels of antibodies to B. pertussis, Haemophilus influenzae* b*, tetanus toxoid, and pneumococcus antigens in healthy mother-infant pairs revealed that the presence of high levels of maternally derived antibodies at birth reduces the response to infant immunization only slightly, therefore supporting the concept of maternal immunization (39). Looking ahead, detailed longitudinal studies integrated with precision vaccinology tools will help identify molecular determinants of human immune response to vaccines.

Peter Richmond (University of Western Australia, Perth, Australia) also highlighted the importance of carefully designed clinical trials to define vaccine efficacy, especially in high-risk populations. For example, in a study based in Papua New Guinea (PNG), a pneumococcal polysaccharide vaccine given to children between the ages of 6 months and 5 years reduced pneumonia mortality (40). However, PNG neonatal or infant schedules of pneumococcal conjugate vaccine are unable to reduce nasopharyngeal pneumococcal colonization rates, an important risk factor for pneumococcal disease, probably because such colonization starts within weeks of birth (41). These observations underscore the importance of considering the target population in vaccine design.

Annette Scheid (Precision Vaccines Program, Brigham and Women’s Hospital/Boston Children’s Hospital, Boston, MA, USA) reported her recently published results on *in vitro* and *in vivo* immune responses elicited by the concurrent administration of HBV vaccine and BCG. Stimulation of whole blood isolated from preterms, newborns, and adults with a combination of BCG and HBV vaccine enhances the production of several cytokines/chemokines, in particular, interleukin-1β (IL-1β), some of them in an age-specific manner. Interestingly, *in vivo* administration of BCG and HBV vaccine to mice enhanced anti-HBV IgG titers in mice immunized on day of life 0 or 7 but not in adult mice (42). These results are of clinical importance because HBV vaccine is often given with BCG as part of the Expanded Program on Immunization.

## SYSTEMS BIOLOGY

At the core of precision vaccinology studies is the use of systems biology tools that can shed new light on molecular requirements for successful vaccination. In the systems biology session, several members of the EPIC-HIPC consortium detailed the various OMICS approaches used and which strategies are used for data integration, storage, and dissemination. As part of EPIC’s goal of defining the molecular ontogeny of early life, Erin Gill (University of British Columbia, Vancouver, Canada) described preliminary transcriptomic results from a pilot cohort of Gambian infants who were receiving immunization at birth or at day of life 7 and who were sampled across the first week of life. *InnateDB* (http://www.innatedb.com/), a publicly available database of the genes, proteins, experimentally verified interactions, and signaling pathways involved in the innate immune response, was used for pathway overrepresentation analysis. Hanno Steen (Boston Children’s Hospital, Boston, MA, USA) and Tue Bennike (Aalborg University, Aalborg, Denmark) highlighted the challenges encountered in applying mass spectrometry to the study of the plasma proteome, especially in newborns and infants. The plasma proteome is highly complex (>5,300 plasma proteins) and exhibits an extreme dynamic range (11 to 12 orders of magnitude) and significant interpersonal variability. A further challenge for plasma proteome analysis is that represented by the relatively small volumes obtainable from newborns and infants. Through optimization of sample preparation protocol and mass spectrometry methodology (43), 199 proteins were quantified in newborn plasma. Interestingly, significant changes were observed as early as 24 h after birth. Joann Arce (Boston Children’s Hospital, Boston, MA, USA) reported the use of metabolomic analyses to characterize the response to BCG *in vitro* and *in vivo*. Several distinct metabolic pathways are modulated by immunization of newborns with BCG, and some of these are also induced by cord blood stimulation with BCG *in vitro*. Scott Tebutt (University of British Columbia, Vancouver, Canada) described a novel analytic approach named *DIABLO* (data integration analysis for biomarker discovery using a latent component method for omics studies) to integrate large-scale molecular OMICS data sets of various characteristics, including transcriptomics, proteomics, and metabolomics. By integrating multiple data sets, this software offers the opportunity to uncover the biological pathways involved in different disease subgroups, thereby identifying biomarkers of disease or of vaccine immunogenicity. These powerful approaches also generate some challenges, including data collection and management, analysis, and integration. Al Ozonoff (Precision Vaccines Program, Boston Children’s Hospital, Boston, MA, USA) described the important role of data management in multidisciplinary systems vaccinology studies. He gave as an example the data management core (DMC) that serves the EPIC-HIPC project. Focused on systems vaccinology of newborns, this project will generate thousands of biological samples to track, process, and analyze. In this context, the DMC is responsible for accurate and reliable data capture, secure data management, project and analytical computing, and quality assurance. To accomplish these important tasks, a cloud computing platform and a decentralized governance structure were chosen, such that EPIC-HIPC cores and projects generate and process data and are also responsible for quality control. Providing cloud-based access to data and integrative analysis tools, the DMC will ultimately be responsible for quality assurance, data access, and public archiving to NIH websites such as *ImmPort* (http://www.immport.org).

James Crowe (Vanderbilt University Medical Center, Nashville, TN, USA) outlined the efforts to characterize the human immunome, that is, the whole set of recombined genes encoding human T cell and B cell receptors. This project will leverage Illumina’s genetic sequencing technologies and is poised to uncover novel diagnostic biomarkers as well as enable the development of highly targeted vaccines and immunotherapies for a wide range of diseases.

William Mohn (University of British Columbia, Vancouver, Canada) reported a study of the impact of early life gut microbiome on asthma severity. Mice treated with vancomycin had an altered gut microbiome and experienced more inflammation in a papain-induced asthma model. Interestingly, administration of a cocktail of short-chain fatty acids (fermentation products of gut bacteria) along with vancomycin had no effect on gut microbiome but reduced inflammation in the same asthma model.

## TRAINED/HETEROLOGOUS IMMUNITY

Vaccines provide pathogen-specific protection, but growing evidence suggests that some vaccines can also exert heterologous (“nonspecific”) effects, potentially protecting against unrelated pathogens. Live attenuated vaccines, including measles vaccine (MV), BCG, and oral polio vaccine (OPV), have positive effects on survival that are substantially greater than those accounted for by a specific pathogen (44). Although this concept is still controversial, due at least in part to an incomplete understanding of the underlying immunological mechanisms, it has received increasing attention. In 2014, the Strategic Advisory Group of Experts (SAGE) of WHO’s immunization program concluded that it is likely that BCG can exert beneficial heterologous effects that are greatest when it is given early in life (45). Peter Aaby (Indepth Network, Guinea-Bissau, Africa/Statens Serum Institut, Copenhagen, Denmark) reported provocative studies suggesting that in some settings administration of nonlive vaccines (e.g., the diphtheria-tetanus-pertussis [DTP] vaccine) may potentially have negative effects for all-cause mortality in female infants. Proposed solutions to this problem include administration of a live vaccine together with or shortly after DTP vaccine (46, 47). This controversial but important area will need further evaluation with respect to scope and underlying mechanisms.

Christine Benn (Indepth Network, Guinea-Bissau, Africa/Statens Serum Institut, Copenhagen, Denmark/University of Southern Denmark, Odense, Denmark) further described the principles of heterologous (“nonspecific”) effects of vaccines. She also reported the results of the Danish Calmette Study, which evaluated the effect of BCG immunization at birth on all-cause and infectious disease hospitalization (48). Interestingly, the beneficial effects of BCG were observed only if the mother had been BCG vaccinated. This might explain the apparently greater effect of BCG immunization in reducing mortality in low-income countries where most of the mothers are BCG vaccinated. This is also in line with results showing that the beneficial nonspecific effects of measles vaccine are strongest in children who had maternal measles antibodies at the time of vaccination (49). Likewise, a literature review suggests that revaccination with live vaccines substantially reduces overall mortality (50). Again, these unexpected and potentially important results challenge current paradigms in vaccinology but require further confirmation as well as investigation of the underlying immunological mechanisms.

Asimenia Angelidou (Precision Vaccines Program, Boston Children’s Hospital, Boston, MA, USA) discussed differences between the BCG formulations produced and given in different locations of the world. She showed that different BCG formulations have different growth properties *in vitro* and induce distinct levels of cytokines in neonatal cord or adult peripheral blood.

Nigel Curtis (The University of Melbourne, Melbourne, Australia) presented the Melbourne infant study, a randomized controlled trial that aims to define whether BCG immunization at birth protects infants from infections while reducing the risk of developing allergies. The scientific basis for linking BCG immunization to allergy development relates to the hygiene hypothesis, in accordance with the idea that exposure to microbes in early life promotes a balanced development of the immune system, thereby reducing risks of allergies and autoimmune diseases (51). That study will also employ systems biology tools to evaluate the immunological mechanisms underlying the heterologous (“nonspecific”) effects of BCG immunization.

## VACCINE ADJUVANTATION AND FORMULATION

An effective vaccine design usually requires proper adjuvantation and formulation systems. While adjuvants may modulate the magnitude and polarization of the immune response, formulation systems may ensure cell- or organ-specific vaccine targeting. Of note, formulation systems can dictate adjuvant properties (e.g., by targeting an adjuvant to the responsive cell subset); therefore, adjuvantation and formulation systems need to be considered in a holistic and intertwined manner. Peter Andersen (Statens Serum Institut, Copenhagen, Denmark) described the adjuvantation system cationic adjuvant formulation 01 (CAF01), which consists of the Mincle agonist TDB (trehalose-6,6-dibehenate), formulated with the cationic liposome-forming surfactant dimethyl dioctadecyl ammonium bromide (DDA), which is internalized by and activates dendritic cells (DCs). CAF01 was developed for generating a BCG booster vaccine and has since demonstrated enhancement of antibody production, germinal center reaction, T cell memory, and Th1/Th17 polarization in several immunization models, including newborn mice. Indeed, CAF01 is now being tested for multiple vaccine indications, including tuberculosis, HIV, malaria, and chlamydia, in phase I clinical trials. In addition, the CAF01 system is amenable to combination with other TLR agonists, such as TLR3 and TLR7/8 agonists (52, 53).

David Dowling (Boston Children’s Hospital, Boston, MA, USA) reported two formulation strategies that enable use of small-molecule TLR7/8 agonists for early life immunization. TLR7/8 agonists robustly activate neonatal DCs but lead to high levels of reactogenicity when delivered in soluble form *in vivo*, therefore requiring appropriate formulation. The first formulation strategy consists of encapsulating TLR8 agonists in polymersome nanoparticles. Encapsulated TLR8 agonists induce robust activation of human and murine neonatal DCs. Moreover, their administration *in vivo* to humanized TLR8 mice with the BCG-specific peptide Ag85B induced an increase in antigen-specific CD4 T cell counts comparable to that seen with BCG immunization (54). The second formulation strategy entails use of a TLR7/8 agonist named 3M-052 possessing a covalent lipid tail that renders it hydrophobic such that it exerts immunogenicity at the site of injection with little systemic absorption. Results obtained in a preclinical model of nonhuman primate immunization at birth demonstrate that 3M-052 admixed with the pneumococcal vaccine PCV13 dramatically enhanced the kinetics and magnitude of generation of antigen-specific Th1 cells and serotype-specific antibody titers, after a single birth dose (55). These results highlight the importance of proper adjuvant selection and formulation to achieve single-dose immunization in early life (55, 56).

Jeffrey Hubbell (University of Chicago, Chicago, IL, USA) presented a new system to target antigen and adjuvant to myeloid cells by coupling them to mannose residues. This complex binds to mannose receptors and enters the endocytic compartment and is therefore particularly well suited to molecules that activate endosomal receptors such as TLR7 agonists [p(Man-TLR7)]. When p(Man-TLR7) is administered in a complex with the experimental antigen ovalbumin [OVAp(Man-TLR7)], it enhances both humoral cell immunity and T cell immunity as well as Th1 polarization. Interestingly, OVAp(Man-TLR7) is more active than OVApMan plus TLR7 (i.e., the TLR7 agonist in soluble form) or OVA plus p(Man-TLR7) (i.e., OVA in soluble form). These observations highlight the importance of targeting both the antigen and the TLR7 agonist to the endosomal compartment. Of note, comparable results were obtained using the circumsporozoite protein (CSP) malaria antigen, supporting the idea of the translational relevance of this formulation system.

## SIGNALING PATHWAYS AND *IN VITRO* MODELING

Some adjuvants included in licensed vaccines target innate immune receptors (e.g., MPL in the human papillomavirus vaccine Cervarix and CpG in the hepatitis B vaccine Heplisav, respectively, activate TLR4 and TLR9). Accordingly, study of innate immune signaling pathways along with the development of proper *in vitro* models that enable their characterization and evaluation of translational relevance is key to identify novel adjuvant targets. Jonathan Kagan (Precision Vaccines Program, Boston Children’s Hospital, Boston, MA, USA) has proposed the provocative concept that not only pathogen-associated molecular patterns (PAMPs) such as that represented by CpG but also specific danger-associated molecular patterns (DAMPs) may exert adjuvant effects. Although the stimulation of DCs with a combination of a PAMP and a DAMP (e.g., lipopolysaccharide [LPS] and ATP) leads to pyroptosis (release of proinflammatory cytokines associated with cell death such as IL-1β), use of oxidized phospholipids (oxPAPC [oxidized 1-palmitoyl-2-arachidonoyl-sn-phosphatidylcholine]) as a DAMP enhances production of cytokines (including IL-1β) without inducing cell death. Accordingly, concurrent *in vivo* administration of LPS and oxPAPC with OVA leads to enhanced T cell activation and polarization compared to administration of LPS or oxPAPC alone (57).

Guzman Sanchez-Schmitz (Precision Vaccines Program, Boston Children’s Hospital, Boston, MA, USA) presented an innovative three-dimensional tissue culture system to model neonatal and adult innate and adaptive immune responses to vaccine formulations. This system consists of a human extracellular matrix covered by endothelial cells. Monocyte migration into the extracellular matrix and back into the medium across the endothelium (reverse transmigration) can differentiate monocytes into dendritic cells. By adding adjuvants or vaccines to this system and allowing monocyte reverse transmigration in their presence and then coculturing vaccine-pulsed monocyte-derived DCs with autologous CD4^+^ T cells, it was possible to model the age-specific prime and boost responses to licensed vaccines such as HBV and BCG. Interestingly, the CD4^+^ T cell responses changed depending on whether newborn plasma or adult plasma was added into the system.

Simon van Haren (Precision Vaccines Program, Boston Children’s Hospital, Boston, MA, USA) presented novel insight into the mechanism of action of an age-specific combined adjuvantation system comprised of an agonist of TLR7/8 together with an agonist of the C-type lectin receptor Mincle. This combination is particularly effective in overcoming impairments in the ability to induce a Th1 response in early life (58). The molecular mechanism was evaluated using quantitative phosphoproteomics, gene expression analysis, and characterization of cellular phenotypes after immunization of newborn mice.

## FUNDING PERSPECTIVE

Representatives from institutions that support vaccine research also presented at the conference. Mercy PrabhuDas (National Institute of Allergy & Infectious Diseases [NIAID], National Institutes of Health, Washington, DC, USA) discussed NIAID programs that support the study of immunity in distinct populations such as the infant and elderly immunity programs as well as new initiatives in immune mechanisms at the mother-fetus interface.

Theodore Schenkelberg (Human Vaccine Project, NY, USA) described the recently established Human Vaccine Project (http://www.humanvaccinesproject.org), a public-private partnership whose goal is to decode the human immune system in order to inform and accelerate vaccine development.

## PANEL DISCUSSION REGARDING THE PATH AHEAD

A panel discussion moderated by Ofer Levy included Peter Andersen, Beate Kampmann, Tobias Kollmann, Stanley Plotkin, Mercy PrabhuDas, Alexander Rumyantsev (Sanofi Pasteur), Ted Schenkelberg, and Geert van den Bossche (Univac, Brussels, Belgium) and considered conclusions from the conference and the path ahead. The Precision Vaccines Conference highlighted the importance of a multidisciplinary approach in translational vaccinology. Novel and powerful systems biology tools enable an unprecedented depth of analysis and may be particularly useful for vaccinology, providing the opportunity to obtain large amounts of molecular and cellular information across the human life span from relatively small biological samples, leading to the following conclusions: (i) vaccine studies should be designed to include appropriate sample collection and storage for downstream systems biology; (ii) computational tools will be critical to convert big data to knowledge; and (iii) once potentially protective signatures are identified, these represent hypotheses that require testing *in vitro* and *in vivo*. Given that the number of potential vaccine formulations with respect to antigen, adjuvant, and formulation is vast and exceeds that realistically evaluable in human clinical trials, *in vitro* systems will be important in providing a means to assess potential reactogenicity and immunogenicity of novel formulations, thereby accelerating and derisking vaccine development. With respect to the studies needed in the years ahead, the panel foresaw the importance of defining the mechanisms by which vaccines engage the human immune system to induce protection by both specific and heterologous (“nonspecific”) effects of live vaccines in rigorously designed studies. Accordingly, the panel highlighted the need for better definitions of how vaccines protect and the relevant differences between distinct but related vaccine formulations that differ in protective efficacy, such as BCG. Opportunities for collaboration include using biomarkers of protection to guide development of novel adjuvantation and formulation strategies poised to usher in a new era in vaccine design, for example, by targeting less-responsive populations at the extremes of life or achieving effective single-shot immunizations that induce durable protection.

Overall, the First International Precision Vaccines Conference highlighted important advancements that are creating new paths for vaccine discovery and design. Important work lies ahead that will benefit from synergistic interaction between academia, funding agencies, government, and industry. In this context, the Second International Precision Vaccines Conference, to be held in 2019 at the Harvard Medical School (Boston, MA, USA), will be at the forefront of these changes by gathering a multidisciplinary community of scientists to review progress and encourage partnerships focused on the most current challenges and opportunities in vaccine research.
